# The management of health care service quality. A physician perspective

**Published:** 2016

**Authors:** L Bobocea, IR Gheorghe, St Spiridon, CM Gheorghe, VL Purcarea

**Affiliations:** * “Carol Davila” University of Medicine and Pharmacy, Bucharest, Romania

**Keywords:** health care service quality, SERVQUAL scale, Seven Prong Model for Improving Service Quality, Health Care Consumer Behavior, perception

## Abstract

Applying marketing in health care services is presently an essential element for every manager or policy maker. In order to be successful, a health care organization has to identify an accurate measurement scale for defining service quality due to competitive pressure and cost values. The most widely employed scale in the services sector is SERVQUAL scale. In spite of being successfully adopted in fields such as brokerage and banking, experts concluded that the SERVQUAL scale should be modified depending on the specific context. Moreover, the SERVQUAL scale focused on the consumer’s perspective regarding service quality.

While service quality was measured with the help of SERVQUAL scale, other experts identified a structure-process-outcome design, which, they thought, would be more suitable for health care services. This approach highlights a different perspective on investigating the service quality, namely, the physician’s perspective. Further, we believe that the Seven Prong Model for Improving Service Quality has been adopted in order to effectively measure the health care service in a Romanian context from a physician’s perspective.

## 1. Marketing Health Care Services

Nowadays, managing marketing in health care services is critically important as the competitive pressure and cost values constantly grow. Because of these changes in the market environment, physicians have to consider solutions to increase the number of health consumers [**1**]. While some managers in health care services prefer to use the conventional above the line advertising methods, others prefer to focus on the management of the services in terms of keeping the existing health care consumers, increase the level of referrals, and generate new ones. The problem with health care services is that patients usually respond to the marketing efforts with hedonic evaluations, so it remains unclear how to approach them. However, in order to be successful or survive on a competitive marketplace, it is essential to deliver to consumers, services that meet or exceed their expectations, suggesting the offer of a high level of service quality. This rule of thumb applies to health care services as well. Lastly, it is of great importance to take into consideration both the opinions of the receipts and the providers, respectively. Therefore, it is required to understand the perceptions of both recipients and providers when shaping and evaluating the concept of service quality.

Many case studies have identified and focused only on the technical characteristic of health care services. More precisely, they concentrated on the physician’s training and skills and on the expected outcome [**2**]. Despite this focus, a massive body of research was conducted to investigate the relationship established between the expected services and the perceived services by health care consumers [**3**,**4**]. Therefore, Services Marketing has developed on the fundamentals of quality evaluation by consumers. A similar process occurs in health care services, namely, the importance of the physicians’ role in shaping the patients’ service expectations, which is hardly included on the list of research objectives. Hence, it may be suitable to have a mixture of elements from both Services Marketing and Health Care Consumer Behavior in order to implement a higher level of service quality. We believe it is also essential to investigate the physicians’ perspectives toward the quality of the delivered services. 

In order to be successful, a health care organization should employ an accurate measurement of health care service quality, as it is necessary to understand the typology and nature of the service delivery system. Without a valid and accurate measure, it is difficult to conceive and implement appropriate strategies for service quality. As such, the most widely used and known scale for the measurement of service quality is the SERVQUAL scale [**3**].

Since the development of the scale, it has been tested in several fields, as for instance, brokerage, banking, utility companies, retail stores, and repair shops. It has also been applied in health [**2**,**5**-**9**], however, the focus was on the perceptions of the health care consumer and scarcely on the physician’s perceived delivery of the health care quality. 

## 2. The concept of service quality in health care services

Service quality is both an abstract and exclusive concept due to its intangibility and inseparability characteristics [**4**]. Services Marketing literature has defined service quality in terms of answers to what and how questions. Specifically, service quality is comprised of what service consumers receive in their interaction with the providers, reflected in technical, physical and outcome quality, as well as technical quality delivery methods, in terms of functional, interactive, and process quality [**3**,**4**]. Parasuraman, Zeithaml and Berry [**3**] pointed out that consumers perceive service quality as the gap between the received service and the expected service. Further, they identified three categories of service evaluation, as it follows [**3**]:

- According to the search properties: consumers may evaluate the service before the purchase. There are two specific dimensions, namely credibility and tangibility. 

- According to the experience properties: consumers may judge service characteristics during consumption or after purchase in terms of reliability, responsiveness, accessibility, courtesy, communications, and empathy.

- According to the credence properties: consumers find it hard to evaluate service characteristics even after purchase or consumption, leading to a higher level of competition.

The quality concept in health care research has been investigated from a different angle. Quality in health care services is “the ability to achieve desirable objectives by using legitimate means” [**10**]. Hence, quality is obtained when a physician contributes accordingly to his patients’ achievable level of health. 

One of the most popular quality assessment models with a specific interest in health care services, except for SERVQUAL scale, has been described by the structure-process-outcome design [**10**]. In this model, the structure indicates the context in which the health care service is provided, the process reflects the way the care is technically delivered, whereas, the outcome suggests the effect of the delivered care on the welfare of the patient. Moreover, in the conceptual design, the service quality was presented as technical in nature and assessed as viewed by the physician. It is acknowledged that physicians pay more attention to the technical and functional dimensions of health care service [**3**,**10**]. 

Returning to SERVQUAL scale, it includes five service quality dimensions, namely, tangibles (the appearance of physical elements), reliability (ability to perform the promised service accurately), responsiveness (promptness and helpfulness), assurance (courtesy, credibility, competence, and customer understanding), and empathy (good communications and customer understanding). There were several other 22 items within each dimension, measured on a seven-point Likert scale, ranging from strongly agree to strongly disagree. Even if, SERVQUAL scale has proved to be robust as an instrument to measure service quality, there was no guarantee that the scale would encompass all dimensions of service quality in all service settings. Consequently, previous studies have reported that SERVQUAL must be modified for each service sector [**7**].

Furthermore, previous tests of SERVQUAL in health care settings revealed mixed outcomes. As such, Babakus and Mangold [**5**] found that SERVQUAL is reliable and valid when employed in a hospital environment. Bowers et al. [**11**] explained that two major additional dimensions were not captured by the SERVQUAL instrument, namely caring and patient outcomes. The caring dimension suggests a “personal, human involvement in the service situation, with emotions approaching love for the patient” and the dimension of outcome dimension, which includes “relief from pain, saving of life, or anger or disappointment with life after medical intervention”. On the other hand, a research conducted by Haywood-Farmer and Stuart [**12**] claimed that SERVQUAL was applied inappropriately since it excluded the dimensions for “core service”, “service customization” and “knowledge of the professional”. In addition, Brown and Swartz [**13**] identified “professional credibility”, “professional competence” and “communications” as significant factors for both the physician and patient, when evaluating service quality. 

Since many studies have been conducted on the perception of health care consumers and service quality, it is also required to have an in depth research on the perception of physicians on service quality. In our conceptual study we believed the perspective of Lee, Delene, Bunda and Kim [**14**] was suitable for the quality in health care services. As such, they modified the SERVQUAL scale, keeping the five dimensions and adding other two, namely “core medical service” [**12**] and “professionalism/ skills” [**6**]. The latter two dimensions were included to measure the technical aspect of the health care service. The seven dimensions are described in **Table 1**. 

**Table 1:** The modified SERVQUAL scale for health care services [**14**]

Dimension	Definition	Authors
Assurance	Courtesy displayed by physicians, nurses, or office staff and their ability to inspire patient trust and confidence	Parasuraman, Zeithaml and Berry [**3**]
Empathy	Caring, individualized attention provided to patients by physicians and their staff	Parasuraman, Zeithaml and Berry [**3**]
Reliability	Ability to perform the expected service dependably and accurately	Parasuraman, Zeithaml and Berry [**3**]
Responsiveness	Willingness to provide prompt service	Parasuraman, Zeithaml and Berry [**3**]
Tangibles	Physical facilities, equipment and appearance of contact personnel	Parasuraman, Zeithaml and Berry [**3**]
Core medical service	The central medical aspects of the service: appropriateness, effectiveness and benefits to the patient	Haywood-Farmer and Stuart [**12**]
Professionalism/ skill	Knowledge, technical expertise, amount of training, and experience	Brown and Swartz [**13**]

**Fig. 1:**
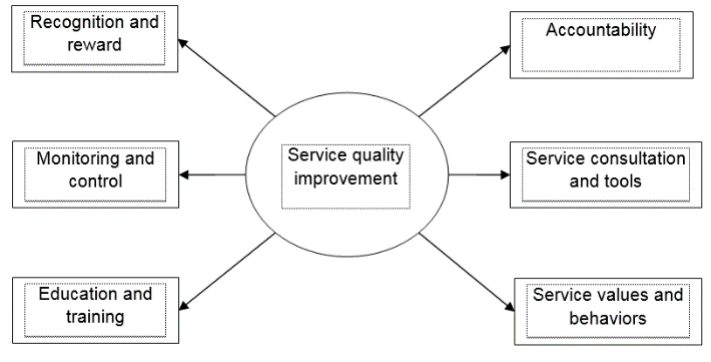
The Seven Prong Model for Improving Service Quality [**15**]

However, if we were to apply the service model proposed by Lee, Delene, Bunda, and Kim [**14**] to the Romanian context, some modifications may occur. Therefore, we have selected the Seven Prong Model for Improving Service Quality elaborated by Kennedy, Caselli and Berry [**15**] because it reflects most of the modified variables in determining health care service quality (**Fig. 1**). 

Prong 1: Accountability for service quality is promoted through the ongoing involvement of multiple layers of leadership.

Prong 2: The service coordinator provides analysis, education, consultation, and training, either at the request of department leaders or as a directive from the CPC. 

Prong 3: Service quality improvement includes setting and communicating service standards, then evaluating performance against those standards. 

Prong 4: Education and training should take place periodically, as frontline employees do not have the skills to understand customers.

Prong 5: Department managers are advised to develop tools and implement processes for ongoing monitoring of service performance.

Prong 6: Teaching desired behaviors and recognizing and rewarding performance elicit more of the desired behavior and contribute to job satisfaction. 
